# Immunophenotypic Profiling of Erythroid Progenitor-Derived Extracellular Vesicles in Diamond-Blackfan Anaemia: A New Diagnostic Strategy

**DOI:** 10.1371/journal.pone.0138200

**Published:** 2015-09-22

**Authors:** Serena Macrì, Elisa Pavesi, Rossella Crescitelli, Anna Aspesi, Claudia Vizziello, Carlotta Botto, Paola Corti, Paola Quarello, Patrizia Notari, Ugo Ramenghi, Steven Robert Ellis, Irma Dianzani

**Affiliations:** 1 Department of Health Sciences, University of Eastern Piedmont, Novara, Italy; 2 Chemical Clinical Analysis laboratory, SCDU, Azienda Universitaria Ospedaliera Maggiore della Carità, Novara, Italy; 3 Department of Public Health and Pediatric Sciences, University of Turin, Turin, Italy; 4 Department of Pediatric Hematology, San Gerardo’s Hospital, Monza, Italy; 5 Pediatric Onco-Hematology, Regina Margherita Children’s Hospital, Turin, Italy; 6 Department of Biochemistry and Molecular Biology, University of Louisville, Louisville, Kentucky, United States of America; European Institute of Oncology, ITALY

## Abstract

Diamond-Blackfan Anaemia (DBA) is a rare inherited anaemia caused by heterozygous mutations in one of 13 ribosomal protein genes. Erythroid progenitors (BFU-E and CFU-E) in bone marrow (BM) show a proapoptotic phenotype. Suspicion of DBA is reached after exclusion of other forms of BM failure syndromes. To improve DBA diagnosis, which is confirmed by mutation analysis, we tested a new approach based on the study of extracellular vesicles (EVs) isolated from plasma by differential centrifugations and analysed by flow cytometry. We chose CD34, CD71 and CD235a markers to study erythroid EVs. We characterised the EVs immunophentoypic profiles of 13 DBA patients, 22 healthy controls and 16 patients with other haematological diseases. Among the three EVs clusters we found, only the CD34+/CD71_low_ population showed statistically significant differences between DBA patients and controls (p< 0.05). The absence of this cluster is in agreement with the low levels of BFU-E found in DBA patients. The assessment of ROC curves demonstrated the potential diagnostic value of this population. We suggest that this assay may be useful to improve DBA diagnosis as a quicker and less invasive alternative to BM BFU-E culture analysis.

## Introduction

Diamond-Blackfan Anaemia (DBA, OMIM 105650) is a rare inherited pure red cell aplasia that typically presents in the first year of life with an incidence of 6–7 newborns per million live births. Penetrance is incomplete and expressivity widely variable even in patients from the same family. Patients with DBA exhibit a macrocytic normochromic anaemia and reticulocytopenia [1]. Approximately 40% of patients display additional clinical abnormalities such as craniofacial, thumb, kidney and heart malformations and growth retardation [2]. Erythroid progenitors (BFU-E and CFU-E) in the patients’ bone marrow (BM) show a pro-apoptotic phenotype and their number is reduced in most patients. The erythrocyte adenosine deaminase (eADA) activity is elevated in 80–85% of patients, but cannot be performed in transfused patients [3].

DBA is considered as the prototype of ribosomopathies. Heterozygous mutations in one of 13 ribosomal protein (RP) genes have been found in about 65% of patients [4]. Haploinsufficiency of ribosomal proteins leads to ribosomal stress and activation of p53–dependent and independent pathways, which result in apoptosis and decreased proliferation [5, 6, 7]. A non-ribosomal form of DBA due to mutations in the GATA1 erythroid-specific transcription factor has also been reported [8, 9].

During the course of the disease, approximately 17% of all DBA patients enter spontaneous or steroid-induced remission, defined as a state of transfusion independence for at least six months with physiologically acceptable haemoglobin levels. The mechanism behind remission remains unknown and about 15% of those who enter remission relapse [3].

Diagnosis of DBA is hampered by the presence of other BM failure syndromes such as Fanconi Anaemia (FA), Shwachman-Diamond syndrome (SDS), Dyskeratosis Congenita (DC) and Transient Erythroblastopenia of Childhood (TEC), which can have overlapping clinical presentations [10]. FA is excluded from a diagnosis by negative results in a chromosome breakage assay while the absence of telomere shortening rules out DC. SDS is characterised by pancreatic insufficiency and often associated with skeletal malformations and neutropenia.

The absence of a unique diagnostic feature for DBA often makes DBA a diagnosis of exclusion. While the identification of the underlying molecular basis for DBA in many patients has now made diagnosis possible through genetic testing, the genes affected in approximately 35% of suspected DBA patients remain unknown leaving a degree of diagnostic uncertainty for these patients. Further confounding a diagnosis of DBA is the increased identification through genetic testing of patients with non-classical forms of DBA including patients with malformations without anaemia or with anaemia presenting as an adult. Recently, we have proposed a rapid and convenient assay readily available in diagnostic laboratories where functional defects in ribosome synthesis linked to haploinsufficiency for large subunit ribosomal proteins could be used as a criterion for making a DBA diagnosis [4]. This approach is currently limited to large subunit ribosomal proteins and would only be supportive by exclusion for DBA caused by defects in non-ribosomal protein genes.

As an alternative strategy for developing a more inclusive assay for possible use in DBA diagnosis we turned to the study of extracellular vesicles (EVs) whose presence may be altered as a consequence of increased apoptosis associated with many bone marrow failures and whose characteristic molecular properties may specifically define the nature of the bone marrow failure. EVs are membrane-bound organelles released by various cell types. Their membrane displays typical markers of the parental cell of origin. Microvesicles (MV) have a diameter of 50-1000nm. MVs have not an endosomal origin and they are enclosed by plasma membrane fragments [11;12]. The outer layer of MV membrane has been often shown to display phosphatidylserine (PS), but this may depend on the cell type from which MVs derive or on the functional cell state [13]. Apoptotic bodies (ABs) are 1–5 μm in diameter. They are released as blebs by cells undergoing apoptosis and they are PS positive.

MVs play a pivotal role in important biological processes such as membrane traffic and horizontal transfer of proteins and nucleic acids among neighboring cells [14]. With regard to the erythroid compartment, it is well known that mature red blood cells shed EVs during eryptosis (a form of erythroid cellular stress) and that reticulocytes eliminate the nucleus and other cellular compartments through vesiculation [15]. No data are available on EV production from erythroid progenitors or early precursors. Classification of EVs, their isolation protocol and detection, molecular details of their release, clearance and biological function are still under intense investigation [16, 17].

In the present study we focused on the immunophenotypic characterization of erythroid EVs from plasma of three different groups of individuals: DBA patients, patients with other haematological diseases, and healthy controls. We reasoned that erythroid EVs may vary in the peripheral blood of DBA patients as a consequence of the loss of erythroid progenitor cells in the marrow of these patients. To our knowledge, this is the first attempt to use EVs in the peripheral blood as an assay for marrow failure and to use EVs as a potential diagnostic tool for Diamond-Blackfan Anaemia.

**Fig 1:**
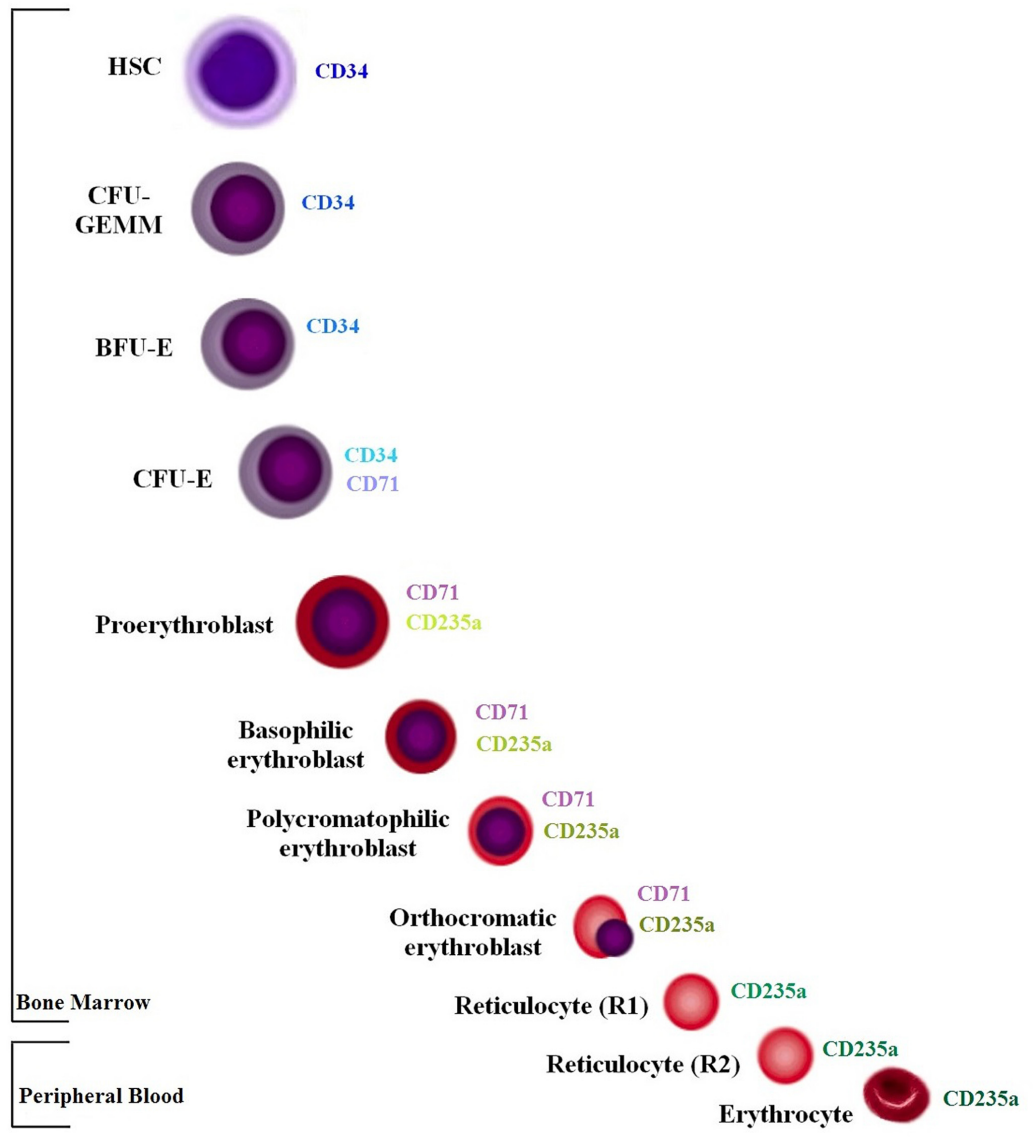
Simplified scheme of erythropoiesis. The most representative markers expressed during erythroid maturation steps are indicated. The intensity colour variation corresponds to expression levels.

Three markers were used to characterise EVs derived from cells of the erythroid lineage: CD34, CD71 and CD235a. CD34 is the main haematopoietic stem cell marker. CD71 is transferrin receptor 1, which is essential for iron uptake and consequently for haemoglobin synthesis during erythroid differentiation (progenitors, i.e. BFU-E, CFU-E and early precursors, i.e proerythroblast, basophilic erythroblast, polychromatophilic erythroblast and orthochromatic erythroblast). Finally, CD235a, i.e. glycophorin A, is the erythroid specific marker and it is expressed by erythroid precursors, i.e proerythroblast, basophilic erythroblast, polychromatophilic erythroblast, orthochromatic erythroblast, reticulocytes and erythrocytes (Fig 1) [18, 19]. Additionally, the presence of PS (e.g a marker of ABs and certain MV types) was tested by Annexin-V binding.

## Methods

### Ethical Statement

The ethical committee of Regina Margherita Children's Hospital (Turin) approved the use of blood samples for diagnostic procedures. A written informed consent was signed by all the individuals under study or their parents, caretakers, or guardians on behalf of the minors/children.

### Patients and controls

Peripheral blood samples were collected from DBA patients (*n = 13)* (Table 1), patients with other haematological diseases (hereafter named non-DBA patients) (*n = 16*) (Table 2) and healthy controls (*n = 22*) (S1 Table). For transfusion dependent patients, peripheral blood samples were collected after 2–5 weeks from the last transfusion, depending on their anaemia severity.

**Table 1:** Clinical characteristics of DBA patients (*patient in clinical remission; **two analysis performed in two independent samples; ^┼^three different analysis a few years apart; ^▲^two different analysis a few years apart). The ranges of PB BFU-E BASAL and BM BFU-E BASAL of healthy individuals are 16.0+/-8.0 and 57.0+/-28, respectively. The normal range of e-ADA is 0,8–1,2 U/g Hb.

DBA PATIENTS	SEX	AGE	AGE AT ONSET	TREATMENT	Hb (g/dL)	MCV (fL)	RBC (10^6/uL)	Reticulocytes (10^9/L)	eADA (U/g Hb)	BM	BM BFU-E BASAL	BM BFU-E WITH SCF	PB BFU-E BASAL	PB BFU-E WITH SCF
**1**	M	24	2 months	TRANSFUSION	8.0	88.8	3.04	13.5	3.2	ERYTHROID APLASIA	0	46	0	1
**2**	F	43	At birth	STEROIDS	10.7	89.8	3.54	N/D	3.9	ERYTHROID HYPOPLASIA	N/D	N/D	1	7
**3**	M	7	1 month	TRANSFUSION	6.8; 6.8**	80.9; 70.8**	2.34; 2.39**	2.8; 5.1**	N/D	ERYTHROID HYPOPLASIA	23	26	1	8
**4**	M	5	1 year	TRANSFUSION	9.5	79.8	3.20	10.7	N/D	ERYTHROID APLASIA	0	0	N/D	N/D
**5**	F	27	2 months	NONE*	10.9	83.8	3.78	45.8	5.3	ERYTHROID APLASIA	0	7	N/D	N/D
**6**	F	4	At birth	TRANSFUSION	9.6	82.1	3.36	6	N/D	ERYTHROID APLASIA	0; 5; 20^┼^	2; 74; 46^┼^	0; 0^▲^	0; 2^▲^
**7**	M	37	1 year	NONE*	11.7	106.8	3.27	35.7	4.1	ERYTHROID HYPOPLASIA	7	41	N/D	N/D
**8**	F	5	6 months	TRANSFUSION	6.2	81.8	2.12	11.6	2	N/D	N/D	N/D	N/D	N/D
**9**	F	17	3 months	STEROIDS	11.5	99.6	3.30	42.8	2.6	ERYTHROID APLASIA	0; 5^▲^	62; 54^▲^	N/D	N/D
**10**	M	13	2 months	STEROIDS	12.6	91.4	4.06	105.0	3.4	ERYTHROID APLASIA	12	89	N/D	N/D
**11**	M	12	2 months	TRANSFUSION	9.0	81.9	3.20	17.7	N/D	NORMAL	N/D	N/D	N/D	N/D
**12**	M	20	1 year	NONE	15.2	89.7	4.8	62.3	5	ERYTHROID HYPOPLASIA	0; 0^▲^	2; 52^▲^	N/D	N/D
**13**	M	25	4 months	TRANSFUSION	10.1	85.6	3.4	45	N/D	ERYTHROID APLASIA	N/D	N/D	N/D	N/D

**Table 2:** Clinical characteristics of Non-DBA Patients.

NON-DBA PATIENTS	SEX	AGE	TREATMENT	Hb (g/dL)	MCV (fL)	RBC (10^6/uL)	Reticulocytes (10^9/L)	DISEASE
**23**	F	7	NONE	13.3	67.6	5.74	44.8	Heterozygous for Beta Thalassemia
**24**	M	4	IRON	12.0	80.5	4.16	69.9	Iron deficiency anaemia
**25**	M	1	NONE	12.2	70.6	4.96	55.7	Heterozygosis for Hb S
**26**	M	5	NONE	9.0	62.3	4.45	47	Iron refractory anaemia
**27**	M	9	TRANSFUSION	9.9	88.0	2.98	33.5	Congenital dyserythropoietic anemia type II (CDA II)
**28**	M	12	NONE	14.4	85.6	4.69	110.5	Spherocytosis (Splenectomized)
**29**	M	20	NONE	17.5	88.9	5.26	80.4	Acquired erytrocytosis
**30**	M	4	NONE	10.8	67.8	4.85	118.1	Iron deficiency anaemia
**31**	F	7 months	NONE	8.2	76.3	3.19	64.9	Homozygous for Beta thalassemia
**32**	M	15	CYCLOSPORIN, STEROIDS	6.7	92.0	2.09	28.9	Aplastic anaemia
**33**	F	8	STEROIDS	9.9	85.4	3.4	44.7	Aplastic anaemia
**34**	F	14	NONE	9.1	85.0	3.19	56.4	Congenital dyserythropoietic anemia type II (CDA II
**35**	M	10	NONE	13.0	95.3	3.86	82.1	Fanconi Anaemia
**36**	M	10	NONE	13.0	77.1	4.97	77.3	Lymphadenitis
**37**	M	12	NONE	13.4	75.4	4.88	N.D	Thrombocytopenia
**38**	M	13	MYCOPHENOLATE	14.7	77.0	5.50	64.6	Autoimmune lymphoproliferative syndrome (ALPS)

### EV enrichment

Blood samples were collected into 3.2% sodium citrate tubes. Platelet free plasma (PFP) was obtained by a centrifugation at 2,400*g* for 10 minutes at room temperature. PFP was then centrifuged at 1,800*g* for 30 minutes at 4°C. Supernatant was subjected to ultracentrifugation at 100,000*g* for 60 minutes at 4°C (Optima^TM^ LE-80K, Beckman Coulter; rotor SW60Ti, Beckman Coulter). Pellets containing EVs were suspended in 1 mL of PBS (filtered using a 0.22 μm pore size membrane) and stored at 4°C.

### EV immunophenotypic profiles

**Fig 2:**
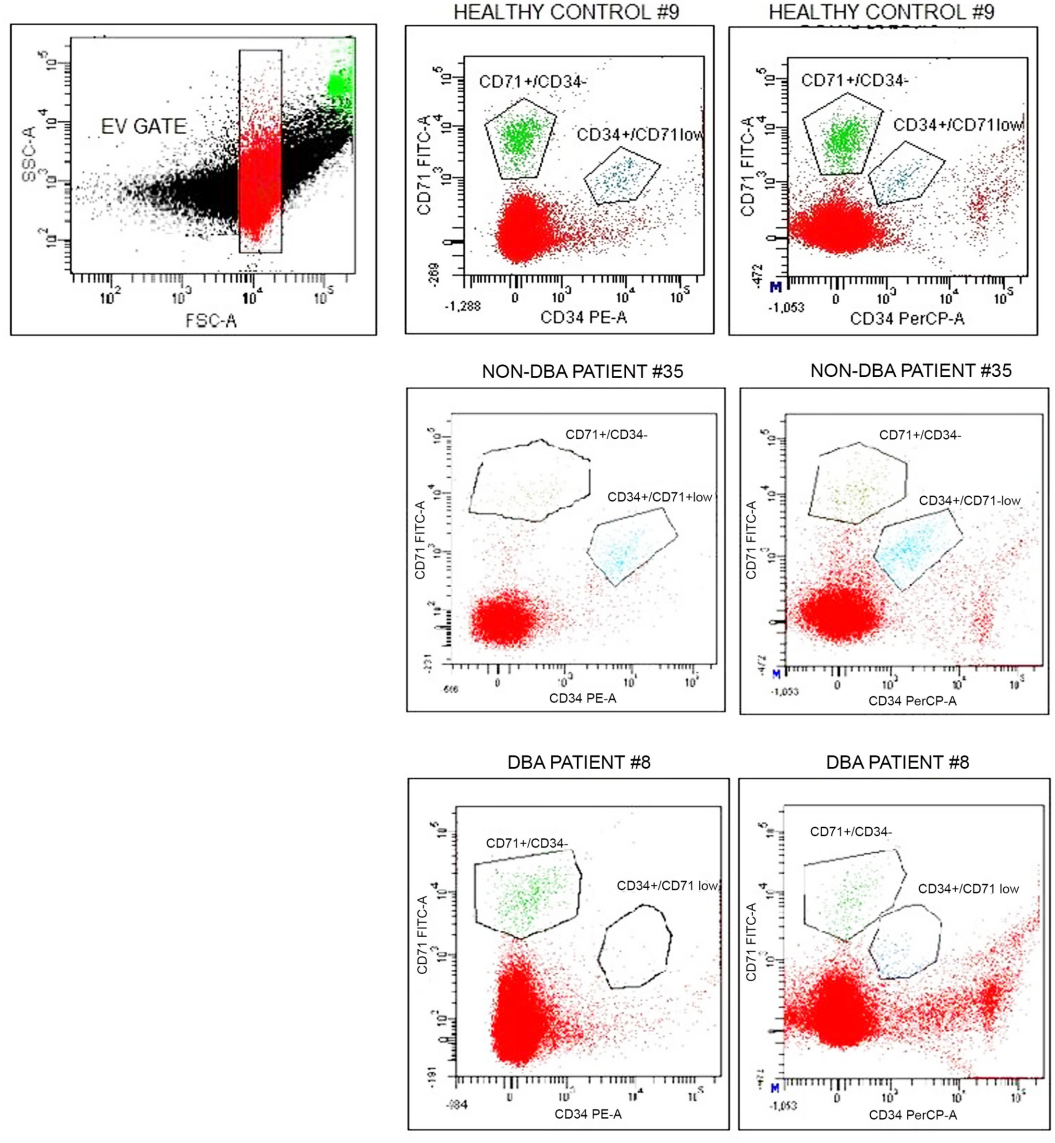
EV distribution in a dot plot graph comparing CD71 and CD34 markers of the events occuring in the EV dimensional gate. CD71+/CD34- (corresponding to CD71+/CD34-/CD235a_low_/PS-) and CD34+/CD71_low_ (corresponding to CD34+/CD71_low_/CD235a+/PS+) populations are shown in representative subjects (healthy controls, DBA patients and non-DBA patients). Comparison of EV populations obtained with or without CD235a staining is shown.

100 μL of resuspended EVs were incubated for 15 minutes at 4°C in the dark with the following combinations of antibodies: 1) anti-IgG2A-FITC/IgG1-PE (isotypic control); 2) anti-CD71-FITC and anti-CD34-PE (this mixture was expected to identify vesicles from BFU-E to ortochromatic erythroblast); 3) anti-CD71-FITC, anti-CD34-PerCP and anti-CD235a-PE (this mixture was expected to identify vesicles from late progenitors to mature erythrocytes). After a washing step with filtered PBS, EVs were resuspended in 400 μL of Annexin-V buffer and 2.5 μL of Annexin-V-APC were added to the mixes 2 and 3. All reagents for the immunophenotypic analysis were purchased from BD. A FACSCanto II flow cytometer (BD) with FacsDiva software (BD) was used for data acquisition. Standard size micro beads of 1 and 2 μm (Flow Cytometry Size Calibration Kit, Invitrogen) were used to calibrate the instrument. We have set the EV dimensional gate to analyse events between 500 nm and 1000 nm. Gate positioning was performed evaluating the median corresponding to 1000nm microbeads and exploiting the direct proportionality between the scattered light and the dimension (Fig 2, upper left panel and S2 Fig).

The relative amount of EVs per samples was determined using the TruCount^TM^ tubes (BD), according to the formula: (number of events in specific gate/ number of TruCount events) * (number of TruCount beads per test/ test volume)* dilution factor. Statistical analyses were performed using Mann-Whitney and Kruskal-Wallis tests.

### ROC curves

Receiver operating characteristic (ROC) curves were established to evaluate the potential diagnostic value of the EV analysis. In a ROC curve the true positive rate (Sensitivity) is plotted in function of the false positive rate (100-Specificity) for different cut-off points of a parameter. The Area Under the ROC Curve (AUC) is a measure of the accuracy of a parameter in the discrimination of two groups under study. An AUC of 1 represents an excellent test; an AUC of 0.5 represents a test that fails to discriminate between the two groups under study. A rough guide for classifying the accuracy of a diagnostic test is the traditional academic point system: 90–100% = excellent, 80–90% = good, 70–80% = fair, 60–70% = poor, 50–60% = fail [20].

## Results

### EV immunophenotypic profile

The markers we used were chosen to identify EVs derived from early progenitors including BFU-E (CD34, CD71), CFU-E (CD71), erythroid precursors (CD71, CD235a) and cells in the late stage of erythroid maturation, i.e reticulocytes and erythrocytes (CD235a) (Fig 1). An isotypic control was used to set the gates of interest (S1 Fig). The immunophenotypic analysis was performed on the events occurring only within the EV dimensional gate (Fig 2, upper panel and S2 Fig).

S1 Fig shows EVs from healthy controls grouped into the following categories: EVs shed from late erythroid progenitors (CD34+/CD71_low_/CD235a_low_/PS+), erythroid precursors (CD71+/CD34-/CD235a_low_/PS-), and cells of late erythroid stages, e.g. reticulocytes and erythrocytes (CD71-/CD235a+/CD34-. About 60% of this population was PS+). Since the anti-CD235a-PE was prone to aggregation we evaluated whether the same EV populations could be defined without using this antibody. Comparison of experiments with and without anti-CD235a antibody showed that CD71+/CD34-/PS- and CD34+/CD71_low_/PS+ populations corresponded to the CD71+/CD34-/CD235a_low_/PS- and CD34+/CD71_low_/CD235a_low_/PS+ populations, respectively (vesicles obtained using or not the antibody against CD235, were counted and their numbers corresponded) (Fig 2, and S2 Table). Consequently, we reasoned that the mixture without CD235a was more suitable for diagnostic purposes because it was not influenced by these technical concerns.

**Fig 3:**
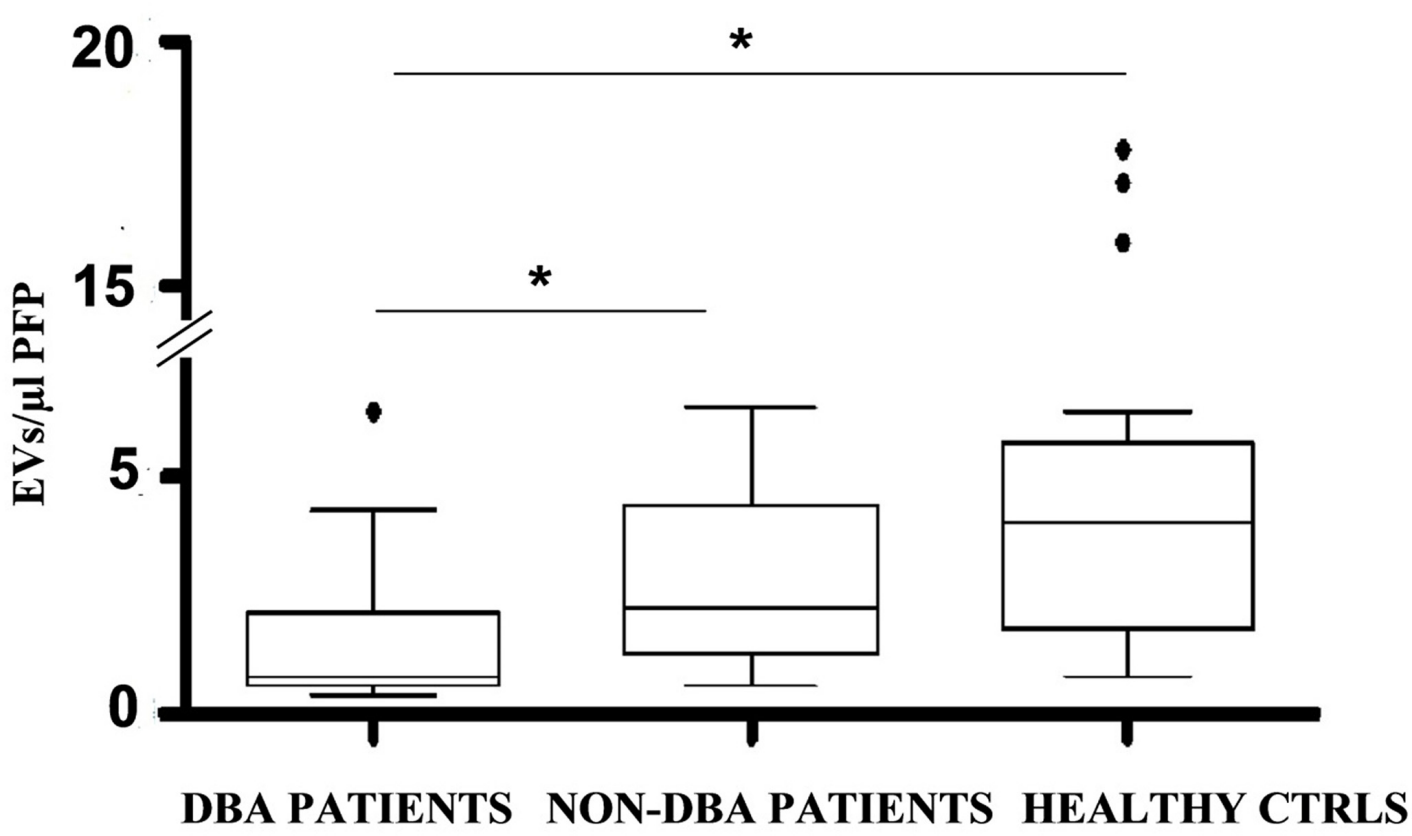
Box plot of Absolute number of events included in the CD34+/CD71_low_ gate. Outliers are shown in black spots. Comparison between DBA patients, non-DBA patients and healthy controls. *The difference of medians is statistically significant (p<0.05, Kruskall-Wallis test).

When we started this work PS was considered as the main marker of EVs. Current data indicate that EVs populations derived from different cells may be PS+ or PS- [15]. In our experiments, its presence was not helpful in the discrimination of EV population derived from DBA patients compared to controls. When we tested DBA patients, only the CD34+/CD71_low_/PS+ population (hereafter named CD34+/CD71_low_) was significantly different when compared to other groups. This population, representing late erythroid progenitors was substantially reduced in 8/13 DBA patients relative to healthy controls. Among patients with other haematological disorders, 15/16 patients showed a proportion of EVs derived from erythroid progenitors similar to controls (Fig 2). The median difference between DBA patients and all the other individuals (non-DBA patients + healthy controls) was statistically significant (p<0.05, Mann-Whitney Test) as was the difference between DBA patients and each of the other groups when compared separately (p<0.05, Kruskal-Wallis Test; Fig 3). As expected, the difference of medians comparing non-DBA patients *vs* healthy controls was not statistically significant (Fig 3).

Finally the CD71-/CD34-/CD235a+ EV population, that is shed from the late stage of maturation, represents the bulk of events. It should be noted that in all transfusion dependent patients, either DBA or non-DBA, this population includes also EVs shed from donor cells. Consequently, we could not compare this population among the three groups analysed.

### ROC curves

**Fig 4:**
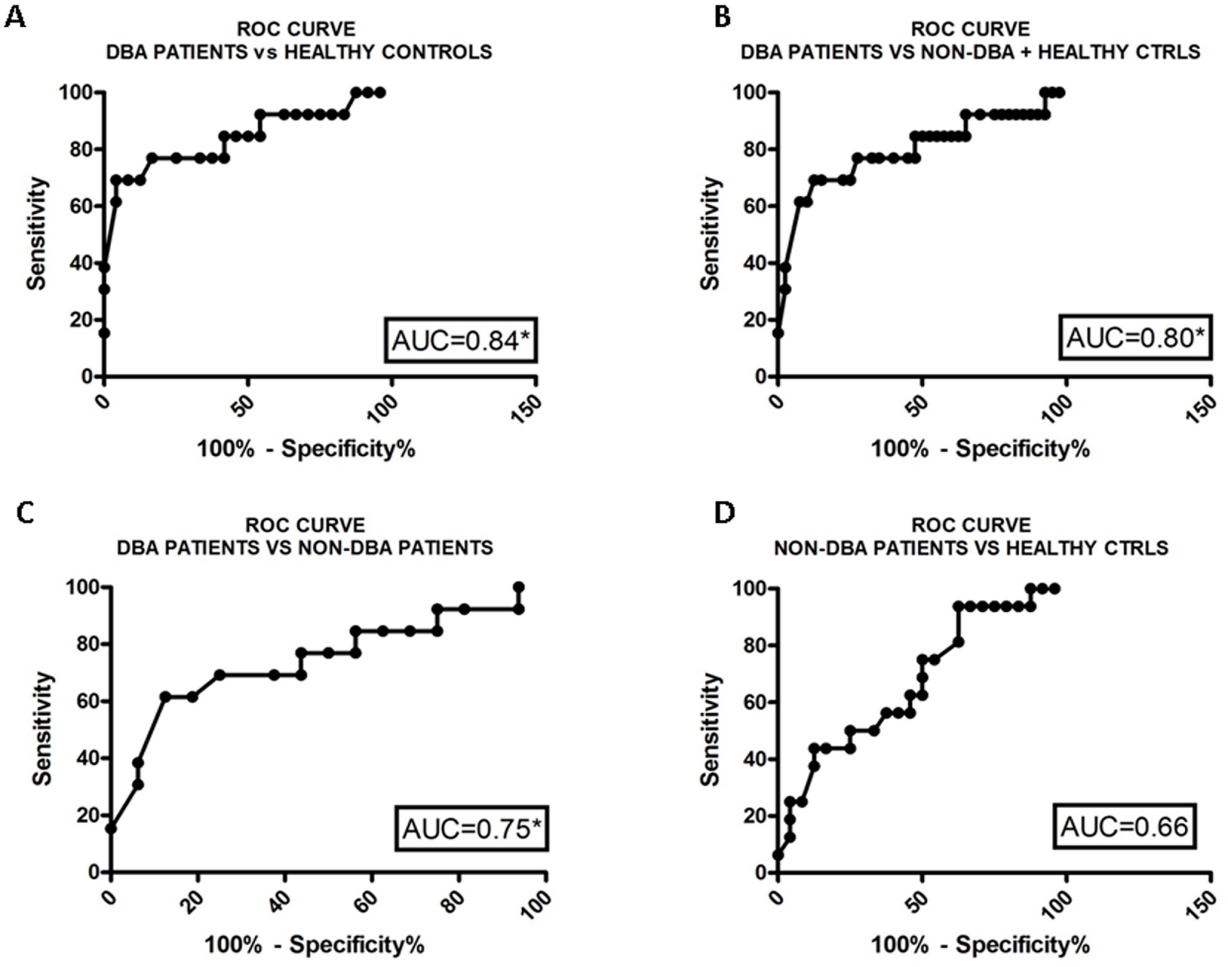
ROC curves analysis of CD34+/CD71_low_ population. ROC curves evaluating the accuracy (AUC) of the CD34+/CD71_low_ analysis in the discrimination of (**A)** DBA patients *vs* healthy controls (**B)** DBA patients *vs* all the others (healthy controls + non-DBA patients) (**C)** DBA patients *vs* non-DBA patients (**D)** non-DBA patients *vs* healthy controls. * p-value <0.01.

The assessment of Receiver Operating Characteristic (ROC) curves demonstrated the potential use of the CD34+/CD71_low_ population as a diagnostic tool. The area under the ROC curve (AUC) that compares DBA patients *vs* healthy controls was 0.84 (= good) while the AUC of DBA patients *vs* all the other individuals was 0.80 (= good). Moreover, comparison between DBA patients and non-DBA patients produced an AUC of 0.75 (= fair). In all comparisons the cluster was able to distinguish the groups under study with a p-value< 0.01. As expected the AUC that plotted non-DBA patients and healthy controls was 0.66 (= poor), consequently this assay cannot discriminate between these groups (Fig 4).

## Discussion

The aim of this study was to evaluate if EV populations in the peripheral blood of DBA patients can be leveraged as a potential diagnostic tool. We studied EVs by flow cytometry, which provides information both on vesicle dimensions and immunophenotypic properties. Vesicles in the size range of 500nm to 1000nm were used in this study. This size range includes a heterogeneous population of EVs including both microvesicles and relatively small apoptotic bodies.

Using our approach we were able to identify EVs ranging from early erythroid progenitors to mature erythrocytes. The bulk of these EVs are likely derived from the peripheral blood, because if the detected EVs derived also from bone marrow EV populations, vesicles derived from all types of erythroid progenitors and precursors would be expected.

Importantly, of the vesicle populations studied, the CD34+/CD71_low_ cluster showed statistically significant differences between DBA patients and healthy controls or patients with other haematological diseases (Fig 3).

The potential diagnostic value of the analysis of CD34+/CD71_low_ EVs was assessed using the ROC curve test. AUC values suggest that this test has a good accuracy to discriminate DBA patients from healthy controls, and/or from non-DBA patients. The CD34+/CD71_low_ EV population is expected to be shed from BFU-E progenitors and our results are in agreement with the low levels of BFU-E found in DBA patients (Table 1).

Actually, this population seems to reflect more the bone marrow BFU-E numbers than the level of anaemia or the disease type. According to this hypothesis, 3 DBA patients (#7, #10, #12), who show this population, had normal erythropoiesis at the time of this analysis (Table 1). In contrast, we found a single DBA patient (#11) with anaemia at the time of this analysis who showed normal levels of this EV population without an evident explanation.

On the other hand, all but one patient with other haematological diseases clustered in the healthy control range. This cluster includes six patients with congenital anaemias characterised by erythroid cell loss at a stage that is later than BFU-E (i.e. HbS, spherocytosis, CDA II and Beta thalassemia), and five patients with acquired conditions. We also analysed a patient with Fanconi Anaemia which can have overlapping features with DBA.

Patient #26 is the only non-DBA patient who did not show this population. He is affected by an iron refractory anaemia with severe microcytosis. We expect that this patient also has a defect of erythroid progenitors, but a BM evaluation has not been performed so far.

Overall these data suggest that the EV assay we devised may be useful to improve DBA diagnosis as a quicker and less invasive alternative to BFU-E cultures. It should be noted that this assay is performed from peripheral blood, is amenable to transfused patients and requires only two working days, whereas BFU-E cultures require 15 working days and needs to be performed using a bone marrow sample in DBA patients.

Finally, this assay could be a useful tool to select erythroid cell-derived EVs in order to study their content and their functional role in erythroid differentiation. It may also be modified to isolate EVs derived by other lineage specific progenitors and used to study other types of bone marrow failure syndromes.

## Supporting Information

S1 Fig EV distribution in dot plot graphs obtained from the acquisition of the samples incubated with anti-CD71-FITC, anti-CD34-PerCP, anti-CD235a-PE and Annexin V-APC. Only the events occuring in the EV dimensional gate were included. Three EV clusters were identified and indicated with different colours.
(TIF)

S2 Fig EV dimensional gating strategy. Beads with two diameters (1 and 2 μm) are shown. The beads were analysed in FSC *vs* SSC plot and in FSC *vs* number of events histogram. The right panel show the application of the EV dimensional gate on a representative control sample. Standard size beads were not intermixed with the sample.
(TIF)

S1 Table Characteristics of healthy controls. (DOC)

S2 Table Absolute number of CD34+/CD71_low_ population obtained from the three groups analyzed without or with CD235a staining, respectively. (*A, B indicate the analysis performed on the same control or patient in two independent samples).
(DOC)
