# Supplementary figures and images for: Immunophenotypic Profiling of Erythroid Progenitor-Derived Extracellular Vesicles in Diamond-Blackfan Anaemia: A New Diagnostic Strategy
Source: PLoS One. 2015 Sep 22;10(9):e0138200. doi: 10.1371/journal.pone.0138200 (PMC4578940; doi:10.1371/journal.pone.0138200)

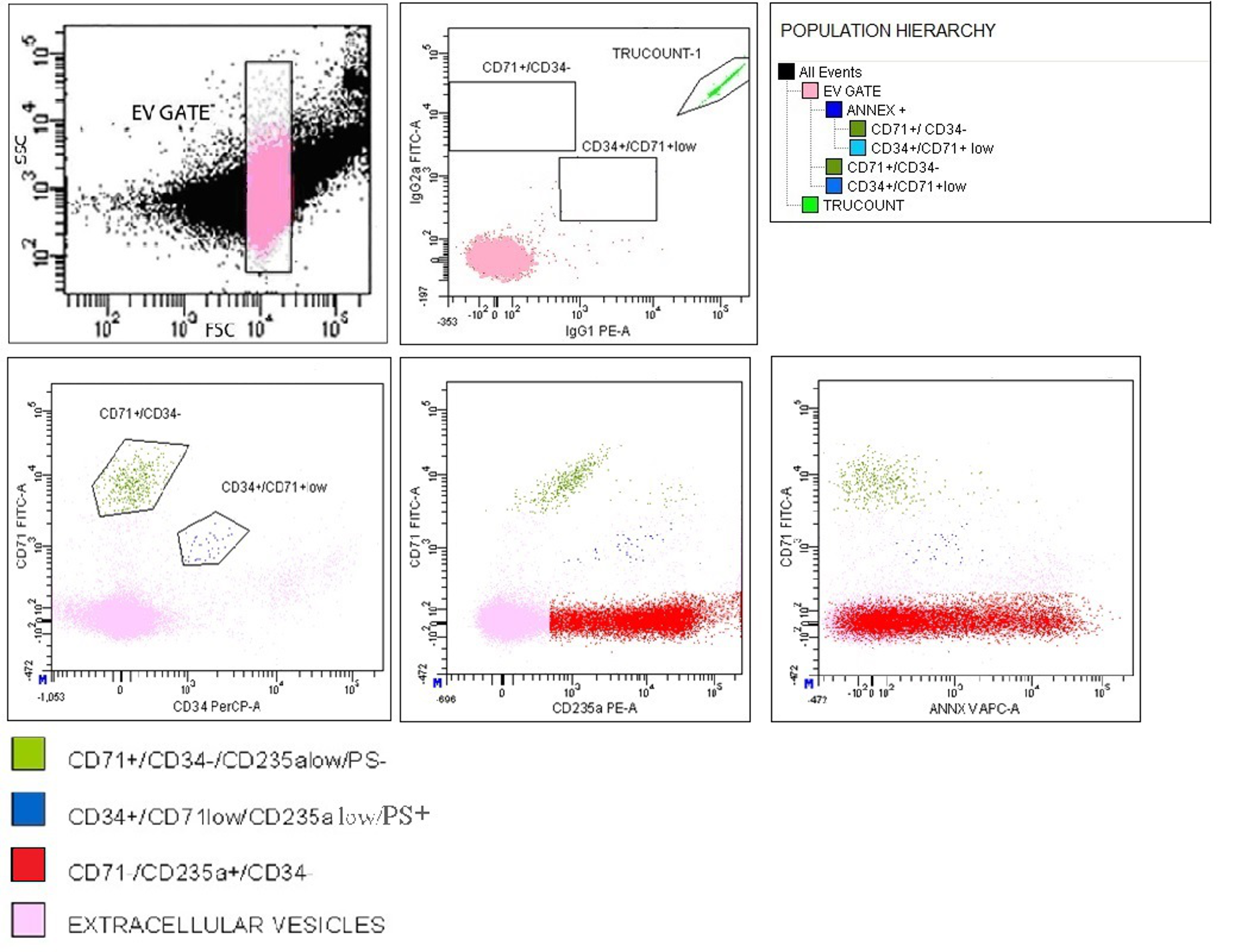

Supplement: S1 Fig — Only the events occuring in the EV dimensional gate were included. Three EV clusters were identified and indicated with different colours. (TIF) [file pone.0138200.s001.tif]

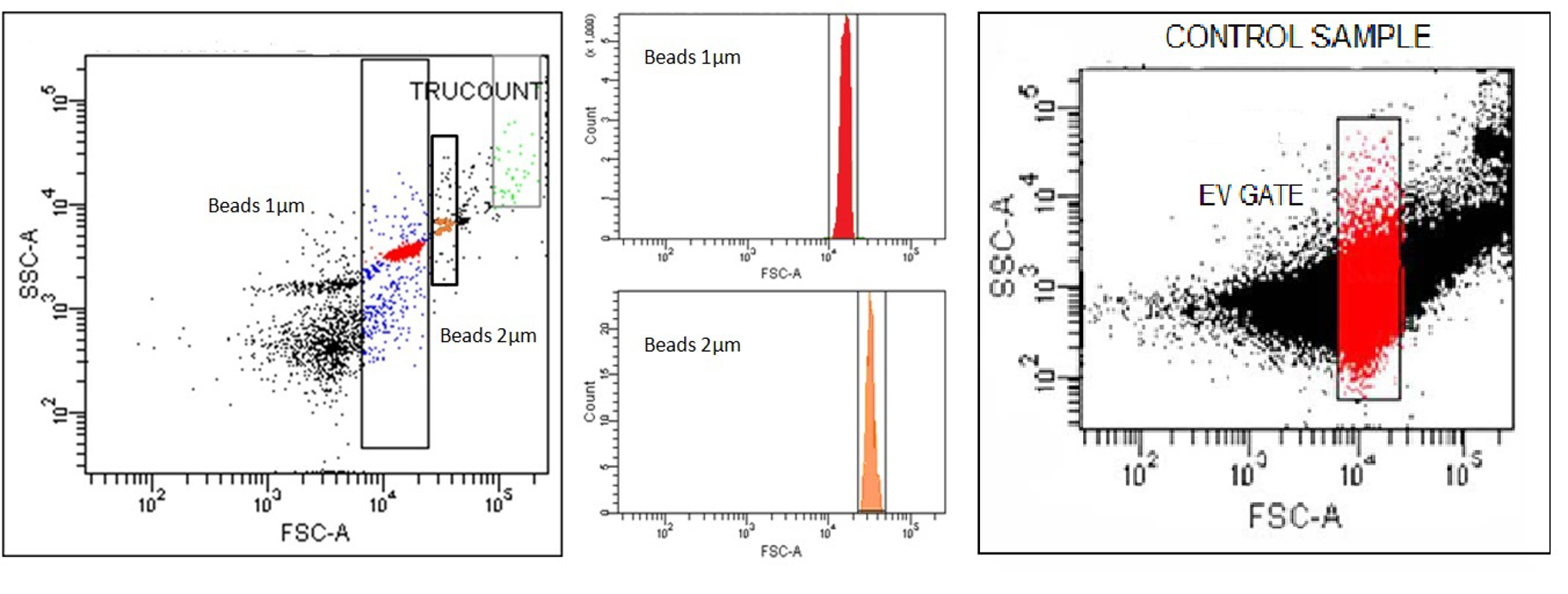

Supplement: S2 Fig — Beads with two diameters (1 and 2 μm) are shown. The beads were analysed in FSC vs SSC plot and in FSC vs number of events histogram. The right panel show the application of the EV dimensional gate on a representative control sample. Standard size beads were not intermixed with the sample. (TIF) [file pone.0138200.s002.tif]
