# Supplementary material for: Immunophenotypic Profiling of Erythroid Progenitor-Derived Extracellular Vesicles in Diamond-Blackfan Anaemia: A New Diagnostic Strategy
Source: PLoS One. 2015 Sep 22;10(9):e0138200. doi: 10.1371/journal.pone.0138200 (PMC4578940; doi:10.1371/journal.pone.0138200)
Supplement: S1 Table — (DOC) [file pone.0138200.s003.doc]

| **HEALTHY CONTROLS** | **SEX** | **AGE** |
| --- | --- | --- |
| 1 | F | 24 |
| 2 | F | 25 |
| 3 | M | 33 |
| 4 | F | 32 |
| 5 | F | 26 |
| 6 | M | 35 |
| 7 | F | 26 |
| 8 | F | 26 |
| 9 | M | 26 |
| 10 | F | 54 |
| 11 | F | 25 |
| 12 | M | 26 |
| 13 | F | 24 |
| 14 | M | 10 |
| 15 | F | 32 |
| 16 | F | 24 |
| 17 | M | 24 |
| 18 | M | 26 |
| 19 | F | 25 |
| 20 | M | 5 |
| 21 | M | 13 |
| 22 | M | 13 |
