# Supplementary material for: Immunophenotypic Profiling of Erythroid Progenitor-Derived Extracellular Vesicles in Diamond-Blackfan Anaemia: A New Diagnostic Strategy
Source: PLoS One. 2015 Sep 22;10(9):e0138200. doi: 10.1371/journal.pone.0138200 (PMC4578940; doi:10.1371/journal.pone.0138200)
Supplement: S2 Table — (*A, B indicate the analysis performed on the same control or patient in two independent samples). (DOC) [file pone.0138200.s004.doc]

| **HEALTHY CONTROLS** | **CD34+/CD71low**  **(without CD235a staining)** | **CD34+/CD71low**  **(with CD235a staining)** |
| --- | --- | --- |
| 1 | 0,8 | 1,0 |
| 2 | 2,6 | 3,0 |
| 3 | 17,8 | 14,4 |
| 4 | 5,9 | 3,2 |
| 5 | 1,6 | 2,8 |
| 6 | 2,2 | 2,8 |
| 7 | 4,9 | 3,4 |
| 8 | 1,7 | 2,4 |
| 9 | 5,2 | 3,1 |
| 10 | 2,2 | 2,0 |
| 11 | 3,8 | 5,4 |
| 12 | 1,3 | 1,0 |
| 13A | 6,2 | 5,3 |
| 13B | 1,4 | 1,7 |
| 14 | 4,2 | 2,8 |
| 15A | 15,9 | 13,2 |
| 15B | 2,9 | 2,5 |
| 16 | 4,5 | 3,1 |
| 17 | 6,4 | 3,5 |
| 18 | 2,4 | 2,6 |
| 19 | 5,3 | 2,5 |
| 20 | 1,7 | 2,1 |
| 21 | 17,1 | 10,4 |
| 22 | 4,5 | 3,6 |
| **NON-DBA PATIENTS** | **CD34+/CD71low**  **(without CD235a staining)** | **CD34+/CD71low**  **(with CD235a staining)** |
| 23 | 6,5 | 4,3 |
| 24 | 4,8 | 3,4 |
| 25 | 1,2 | 2,4 |
| 26 | 0,6 | 1,3 |
| 27 | 3,6 | 4,3 |
| 28 | 1,4 | 1,5 |
| 29 | 2,1 | 3,1 |
| 30 | 1,5 | 2,1 |
| 31 | 2,4 | 3,6 |
| 32 | 4,8 | 4,4 |
| 33 | 0,8 | 1,3 |
| 34 | 1,1 | 0,7 |
| 35 | 4,0 | 4,9 |
| 36 | 1,4 | 1,9 |
| 37 | 4,1 | 3,2 |
| 38 | 4,5 | 4,9 |
| **DBA PATIENTS** | **CD34+/CD71low**  **(without CD235a staining)** | **CD34+/CD71low**  **(with CD235a staining)** |
| 1A | 0,7 | 0,6 |
| 1B | 0,4 | 0,8 |
| 2 | 0,7 | 0,8 |
| 3 | 0,8 | 1,2 |
| 4 | 0,8 | 0,8 |
| 5 | 0,4 | 0,6 |
| 6 | 0,4 | 0,9 |
| 7 | 1,6 | 1,2 |
| 8 | 0,8 | 0,7 |
| 9 | 0,6 | 0,6 |
| 10 | 4,3 | 2,6 |
| 11 | 1,2 | 1,4 |
| 12 | 6,4 | 4,3 |
| 13 | 2,7 | 3,6 |
